# Vaccines Against Hantavirus Infections: A Systematic Literature Review

**DOI:** 10.3390/vaccines14100898

**Published:** 2026-10-13

**Authors:** Giovanni Cioni, Giovanna Letizia Calò, Gianmarco Imperiali, Lamberto Manzoli, Maria Elena Flacco

**Affiliations:** 1 Department of Environmental and Prevention Sciences, University of Ferrara, Via Fossato di Mortara 64, 44121 Ferrara, Italy; giovannaletizia.calo@unife.it (G.L.C.); mariaelena.flacco@unife.it (M.E.F.); 2 Department of Medical and Surgical Sciences, University of Bologna, Via San Giacomo 12, 40138 Bologna, Italy; gianmarco.imperiali2@studio.unibo.it (G.I.); lmanzoli@post.harvard.edu (L.M.)

**Keywords:** hantaviruses, hantavirus infection, vaccines, zoonotic viruses, clinical trials, systematic review

## Abstract

**Background/Objectives:*** Hantaviruses* infections are responsible for a hemorrhagic fever with renal and cardiopulmonary syndrome with case-fatality rates up to 50%. While inactivated vaccines are already available in China and South Korea, several additional candidate vaccines are under development. This systematic review summarizes the current clinical evidence on immunogenicity and short-term safety of vaccines targeting different hantavirus strains. **Methods:** Following PRISMA 2020 guidelines, a systematic literature search was carried out to identify randomized and nonrandomized trials of any phase. Data were synthesized narratively within strata defined by vaccine platform, target virus, assay, immunogenicity and safety. **Results:** Ten trials on 961 adults were included: four single-arm trials on the inactivated vaccine and six early-phase DNA-vaccine trials. One month after last dose, the inactivated vaccine achieved neutralizing seropositivity by PRNT_50_ in 45.1% of the tested subjects after two priming doses and 55.7% after three, while the corresponding binding-antibody values measured by IFA were 87.3% and 95.7%. Among DNA vaccines, homologous responses reached 100% against HTNV and 83.3% against PUUV with monovalent constructs, whereas dual responses to the bivalent construct were registered in 33.3% and 77.8% of the subjects, when measured through PRNT_50_ and PsVNA_50_, respectively. One serious adverse event occurred, most likely unrelated to vaccination. **Conclusions:** No serious safety signal was identified, although safety remains insufficiently characterized. Measured immunogenicity depends as much on the assay as on the vaccine, and, in the absence of a validated correlate of protection, it cannot be read as a clinical benefit. Future research should prioritize validated correlates of protection and innovative trial designs adapted to low-incidence diseases.

## 1. Introduction

*Hantaviruses* are a group of enveloped, negative-stranded, RNA viruses, primarily carried by rodents, and potentially transmitted to humans through inhalation of aerosols contaminated with feces, urine, or saliva of infected mice [1,2,3,4]. Human infections are relatively uncommon: each year, around 200,000 cases are documented worldwide [5], characterized by a defined geographical pattern, and differing in etiology, clinical manifestations, and available prevention strategies [5,6,7]. In East Asia, which carries the largest burden of cases, hantavirus infections are caused by the so-called Old-World viruses (such as *Hantaan* and *Puumala* strains), which are responsible for a hemorrhagic fever with renal syndrome (HFRS), showing a case-fatality rate ranging from 5% to 15%, according to the available estimates [7]. The so-called New-World viruses, such as *Andes* and *Sin Nombre* strains, are responsible for outbreaks mainly recorded in the Americas, and in Northern and Central Europe [1]. The *Andes* strain (so far, the only one capable of inter-human transmission [8,9] and the other New-World viruses are responsible for hantavirus cardiopulmonary syndrome (HCPS), affecting 70–80% of infected individuals [10,11], and carrying a case-fatality rate of up to 50% [7,12].

To date, no specific therapy exists for HFRS and HCPS [13]; thus, vector control strategies and immunization campaigns for high-risk individuals (such as military force personnel, agricultural workers and forestry workers and for those living in epidemic areas [14] have been the cornerstone of preventive policies [15]. Whole-virus inactivated vaccines against Old-World viruses have been approved in China and South Korea for adults aged 16–60 years, and extensively administered since 1995 [7,13]. Conversely, no licensed vaccines are currently available for infections caused by New-World strains, and, so far, only data from phase 1 clinical trials are available [15]. If, on one hand, the antigenic diversity among hantavirus species may create technical challenges for vaccine development [6,13], the lack of an efficacious vaccine is likely a consequence of the limited and geographically scattered number of cases, which causes difficulties in identifying a proper region to run a classic phase III efficacy trial and limits commercial attractiveness, as the market would inevitably be restricted [15].

In this scenario, the recent Hantavirus *Andes* outbreak, with 13 cases and three deaths among the 149 passengers and crew of the MV Hondius cruise ship, has posed several challenges in terms of public health preparedness and response toward a zoonotic disease almost neglected [8]. Additionally, the outbreak has revealed some knowledge gaps in terms of transmission dynamics, risk of infection, virus isolation, and quarantine procedures [8,16]. Although it is likely that a new boost towards vaccine development against New-World strains will come from the recent events, the available literature data on the topic are sparse and focused on licensed vaccines. Thus, the aim of the present systematic literature review is to summarize the available evidence from interventional clinical studies on two outcomes: (i) the humoral immunogenicity of hantavirus-targeted vaccines, reported separately as assay-specific seropositivity and assay-specific seroconversion; and (ii) the short-term safety and reactogenicity of vaccines targeting the different hantavirus strains.

## 2. Materials and Methods

### 2.1. Search Strategy and Selection Criteria

The reporting of this systematic review followed the Preferred Reporting Items for Systematic Reviews and Meta-Analyses (PRISMA) 2020 statement (completed checklist in Table S5) [17]. Clinical trials, enrolling healthy individuals of any age, were considered eligible if they had a randomized or nonrandomized interventional design, including both single-arm and multi-arm studies, and if they evaluated immunogenicity and/or short-term safety after administration of a hantavirus-targeted vaccine, and were published in English. Any vaccine platform was considered eligible, including inactivated whole-virus vaccines and DNA vaccines, regardless of the stage of clinical development. Studies conducted exclusively in animal models or in vitro were excluded, as were observational studies without an interventional vaccination component. Trials focused only on bioequivalence or pharmacokinetic outcomes were also excluded when immunogenicity or safety data were not reported and could not be obtained from investigators. Two authors (G.C. and G.L.C.) independently searched PubMed, EMBASE, Scopus and ClinicalTrials.gov from inception to 23 September 2026, using various combinations of the following search terms: (“Hantavirus” OR “HFRS” OR “Hemorrhagic fever with renal syndrome” OR “HCPS” OR “Hantavirus cardiopulmonary syndrome”) AND “vaccin*” AND “Human*”. While maintaining a common overall architecture, several alternative strings were used after adjustment for each database, and the full strategies are reported in the Supplementary Materials, Table S1. The reference lists of reviews and retrieved articles were also searched. Two authors (G.C. and G.L.C.) independently performed title/abstract screening, full-text eligibility assessment, data extraction and methodological appraisal. Disagreements were resolved by discussion and, where unresolved, arbitrated by a third senior author (M.E.F.). The methodological quality of the included studies was assessed using risk-of-bias tools appropriate to each study design. Randomized trials were appraised with the revised Cochrane risk-of-bias tool for randomized trials (RoB 2) [18] across its five domains, and an overall judgement of *low risk*, *some concerns* or *high risk* was assigned. Nonrandomized trials were therefore evaluated with the NIH Quality Assessment Tool for Before–After (Pre–Post) Studies With No Control Group [19], independently by two authors, with item-level judgements and supporting explanations in Table S4.

### 2.2. Data Extraction

For each included trial, two authors independently extracted the following information:

(a) Vaccine construct, dosage and schedule, route and delivery device, and funder;

(b) General characteristics of the study: trial registration identifier, design, country and number of centers, phase, and participants enrolled;

(c) Characteristics of the compared arms and timing of outcome assessment;

(d) Immunogenicity outcomes, which, across the included studies, were reported in terms of (I) seropositivity and/or (II) seroconversion rates. In this scenario, the immunogenicity outcomes mentioned above were defined as follows: (I) **Seropositivity** is the proportion of participants whose post-vaccination titer reaches or exceeds an assay-specific threshold at a given time point, irrespective of the baseline value [20,21]; (II) **seroconversion** requires a defined change from baseline: a post-vaccination titer ≥ 40 when the day-0 titer was <20, or a ≥4-fold rise when the baseline titer was ≥20 [22,23,24,25]. Seropositivity and/or seroconversion data were extracted at the nearest post-vaccination time point, usually one month after completion of the primary schedule or booster dose. Where a trial reported only one of the two endpoints, this is stated in the tables, and the other is not imputed.

The three assays used across the included trials measure different quantities [26,27,28].

- The 50% plaque reduction neutralization antibody test (PRNT_50_), defined as the highest serum dilution capable of reducing at least by 50% the average number of plaques observed in the virus-only control plates [23];
- The 50% pseudovirion neutralization assay (PsVNA_50_) measures the neutralization of an engineered surrogate particle and systematically yields higher titers than PRNT_50_ in the same sera;
- The immunofluorescence antibody assay (IFA) detects binding antibody and does not measure neutralization; IFA results are therefore reported as a separate, non-functional endpoint and excluded from every statement about neutralizing responses. Results obtained with different assays were not included in the present review.

(e) Vaccine efficacy, when reported, was evaluated from 30 days after completion of a two-dose primary immunization course until the end of follow-up and was defined as a virologically confirmed hantavirus infection. Confirmation was based on detection of hantavirus-specific immunoglobulin M, rising titers of hantavirus-specific immunoglobulin G, detection of hantavirus-specific RNA by polymerase chain reaction in clinical specimens, or detection of hantavirus antigen by immunohistochemical analysis [10].

(f) Short-term safety outcomes, including the main adverse events (AEs) reported after immunization, separately considering serious adverse events (SAEs) and mild-to-moderate adverse events.

## 3. Results

### 3.1. Characteristics of the Included Studies

**Figure 1:**
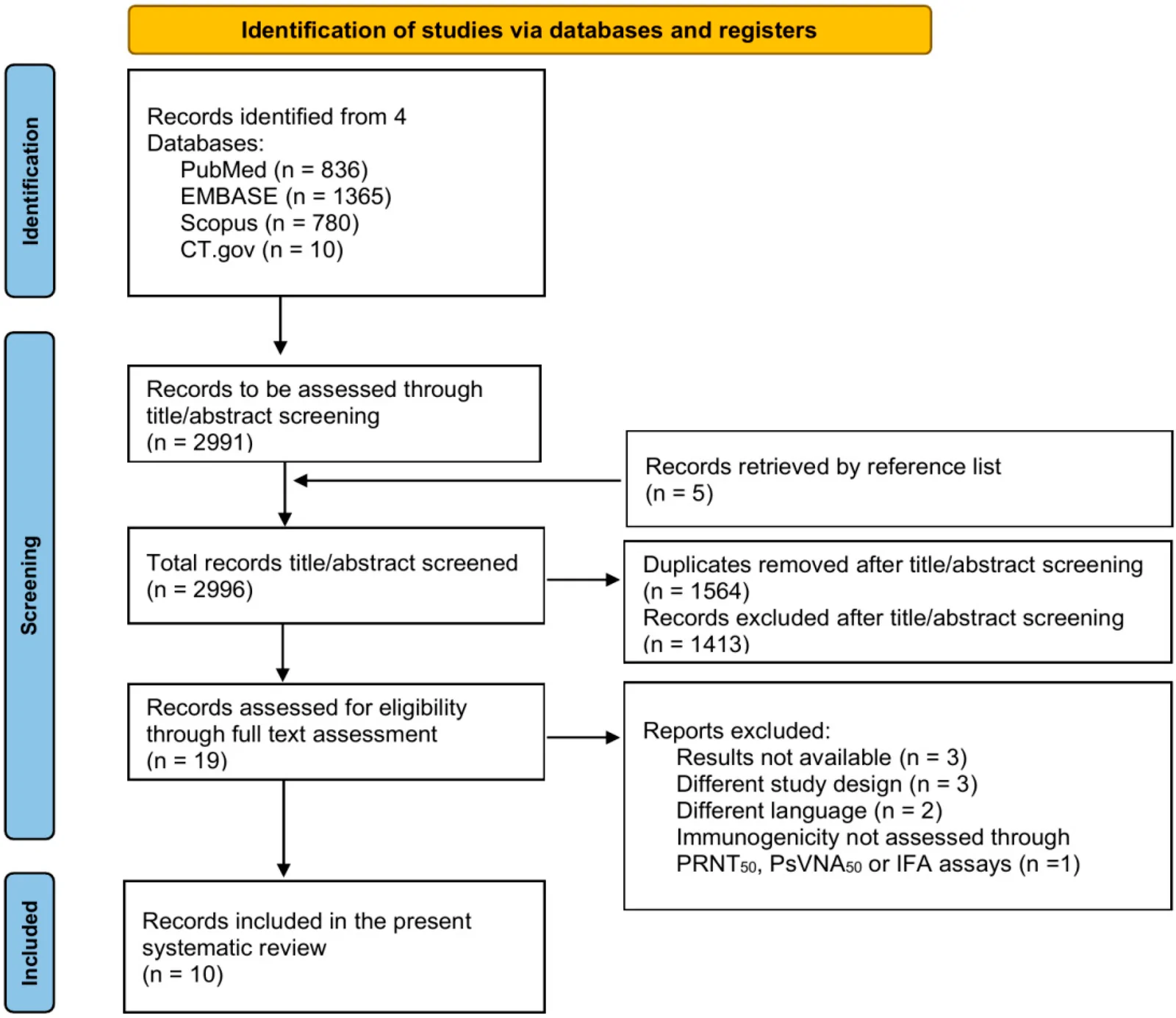
Prisma 2020 flow diagram of study identification, screening and inclusion.

The initial search identified 2996 records retrieved from different databases: 836 records from PubMed, 1365 from EMBASE, 780 from Scopus and 10 from ClinicalTrials.gov (2991 records), with five further records identified from reference lists (Figure 1). After duplicate removal and title/abstract evaluation, a total of 19 studies were assessed for eligibility; of these, ten trials [20,21,22,23,24,25,29,30,31,32], enrolling a total of 961 participants, met the inclusion criteria and were included in the review. The main general characteristics of the included trials are summarized in Table 1a. No registration identifier was reported or retrieved for three trials [29,31,32]. One trial [30] is a completed phase 1 trial whose results are available only on ClinicalTrials.gov (accessed on 23 September 2026). Another trial [31] reported immunogenicity data only and was included accordingly. Study intervention characteristics, such as vaccine allocation, the number of doses, and dosing schedules and delivery methods, are reported in Table 1b. The nine excluded studies and the corresponding reasons for exclusion are listed in Table S1

The studies identified in this review exhibit significant heterogeneity in vaccine type and characteristics. Nine included trials [20,21,22,23,24,25,29,30,31] enrolled healthy adult volunteers (age range: 18–75 years); one [32] enrolled agricultural workers in an endemic country. Four trials were conducted in South Korea [20,21,31,32]; two early-phase (unspecified) [31,32] and two phase III trials [20,21] evaluated Hantavax^®^, an inactivated whole-virus vaccine directed against HFRS caused by the Hantaan strain. Five phase I trials and one phase IIa trial were conducted in the United States [22,23,24,25,29,30] and evaluated different schedules and dosages of DNA plasmid vaccines expressing the envelope glycoprotein genes of Hantaan (HTNV), Puumala (PUUV) or Andes (ANDV) viruses. These vaccines were either single-strain [22], or combined to obtain a HTNV/PUUV vaccine mixture [23,24,25,29,30]. Five of six trials were randomized [22,23,24,25,30]; one was a nonrandomized, open-label study [29]; only two trials included a placebo comparator [22,24]. Primary dosing regimens generally adhered to a three-dose prime-boost sequence (typically administered on days 0, 29, and 169). However, a booster dose was frequently planned to optimize neutralizing antibody titers. Despite the variability in delivery schedules, outcome measures remained standardized across all trials, focusing on the core pillars of safety and immunogenicity.

Figure 1 reports separately the records identified from each database and registry, the records retrieved from reference lists, duplicates removed, records excluded at title/abstract screening, reports assessed in full text and reports excluded with reasons, and the studies included. No automation tool was used during the selection process. It was adapted from the PRISMA 2020 statement [17], licensed under CC BY 4.0.

### 3.2. Immunogenicity Outcomes

The included trials showed variable immune responses for both the inactivated whole-virus vaccine and the DNA vaccines. Table 2a–c show the reported seropositivity and/or seroconversion rates, assessed using the different laboratory assays mentioned above. Since no validated correlate of protection exists for hantavirus infection in humans, neither a titer above an assay threshold nor a rise from baseline indicates protection against infection or disease. Results are presented in separate strata by vaccine platform, target virus, assay, and outcome definition; values obtained with different assays are not compared, and IFA results are reported separately from PRNT_50_ and PsVNA_50_ results.

#### 3.2.1. Inactivated Whole-Virus Vaccine (Hantavax^®^)

Three single-arm trials evaluated Hantavax^®^ reported seropositivity, and one reported seroconversion (Table 2a) [20,21,31,32]. Longitudinal data showed that, one month after completion of a two-dose and one-booster regimen, seropositivity measured by PRNT_50_ was 50.0% (7/14) in the earliest trial and 45.1% (64/142) in the 2016 phase III trial [20,32], whereas the seropositivity rate measured by IFA was higher at 87.3% (124/142) [20]. After a three-dose primary course plus a booster, the 2020 phase III trial [22] reported seropositivity rates of 55.7% by PRNT_50_ and 95.7% (266/278) by IFA [21]. The remaining trial [31] reported seroconversion rather than seropositivity, after two doses and without a booster, and found a PRNT_50_ seroconversion rate of 33.3% (10/30).

#### 3.2.2. DNA Vaccines: HTNV and PUUV Constructs

Five trials [23,24,25,29,30] evaluated HTNV and PUUV DNA constructs, with doses, delivery devices and schedules that differ in every trial (Table 1b). In the earlier phase I trial [29], seropositivity was 30.0% (3/10) against HTNV and 44.4% (4/9) against PUUV in the monovalent arms, while the bivalent arm gave 44.4% (4/9) against HTNV, 33.3% (3/9) against PUUV and 22.2% (2/9) against both viruses one month after the third dose. In the second [25], which raised the dose and delivered it by intramuscular electroporation, seropositivity was 63.6% (7/11) against HTNV and 75.0% (6/8) against PUUV in the monovalent arms, and 33.3% (3/9) against HTNV and 77.8% (7/9) against PUUV in the bivalent arm, with the dual rate not reported.

The phase IIa trial [24] reported only the proportion responding to both viruses, measured by PsVNA_50_, and did not report PRNT_50_ one month after the booster dose on d197. Dual seropositivity was 76.7% (23/30), 73.3% (22/30), 80.0% (24/30) and 66.7% (20/30) in arms 1 to 4, respectively. The two arms receiving the full primary course (arms 1 and 3) performed better than the two in which one priming dose was replaced by placebo (arms 2 and 4) at both dose levels, whereas doubling the antigen dose produced no consistent gain.

The 2024 phase I trial (24] is the only one reporting both assays for both endpoints in every arm one month after the booster dose at d194. Homologous seropositivity in the monovalent arms was 100% (7/7) against HTNV and 83.3% (5/6) against PUUV by PRNT_50_, and 100% (7/7) and 100% (6/6), respectively, by PsVNA_50_. Cross-reactive seropositivity against the virus not contained in the construct was 42.9% (3/7) against PUUV in the HTNV arm and 33.3% (2/6) against HTNV in the PUUV arm by PRNT_50_, but 100% (7/7) and 50.0% (3/6) by PsVNA_50_. In the bivalent arm, seropositivity was 44.4% (4/9) against HTNV and 77.8% (7/9) against PUUV by PRNT_50_, and 77.8% (7/9) and 88.9% (8/9) by PsVNA_50_. Seroconversion in the same trial (Table 2c) follows the same pattern. Dual seroconversion against both viruses was 42.9% (3/7), 33.3% (2/6) and 33.3% (3/9) in arms 1 to 3 by PRNT_50_, but 57.1% (4/7), 66.7% (4/6) and 77.8% (7/9) by PsVNA_50_. In the bivalent arm, the apparent dual-response rate therefore more than doubles, from one third to more than three quarters, depending on which assay is used on the same sera.

The registry-only trial [30] is the only one comparing intradermal with intramuscular electroporation directly, and its six arms are small and unequal. By PsVNA_50_, homologous seropositivity was 100% (4/4) with 0.6 mg of HTNV intradermally and 75% (9/12) with 3.0 mg intramuscularly; for PUUV it was 75% (3/4) intradermally and 33.3% (4/12) intramuscularly. The two bivalent arms enrolled two participants each and gave 0% (0/2) and 50% (1/2). Seroconversion rates were 100% (4/4), 50% (6/12), 75% (3/4), 66.7% (8/12), 0% (0/2) and 50% (1/2).

#### 3.2.3. DNA Vaccine: ANDV Construct

One phase I trial evaluated a monovalent ANDV construct in four arms, comparing two dose levels with two schedules [22]. One month after the booster, seropositivity by PsVNA_50_ was 55.6% (5/9) in the 2 mg arm receiving one placebo priming dose and 100% (9/9), 88.9% (8/9) and 88.9% (8/9) in the remaining three. Seroconversion rates across the same four arms were 55%, 88%, 60% and 89%, with numerators and denominators not reported. The two arms receiving the complete four-dose course reached the highest values at both dose levels, so here too, the schedule rather than the antigen dose separates the arms. PRNT_50_ results were not reported for either endpoint. This is the only trial of a vaccine directed against a New-World hantavirus and it is a first-in-human study.

### 3.3. Safety Outcomes

The safety profile was assessed in nine of the ten included trials (Table 3) [20,21,22,23,24,25,29,30,32]. One trial did not report any safety information and was excluded from this analysis [31]. Solicited AEs were recorded 7 to 14 days after each injection, and longer-term surveillance (30 to 365 days after the final dose) captured any delayed-onset serious events. Across a safety population of 919 participants, a single serious adverse event was recorded, in the registry-only trial NCT03718130 [30]: a case of alcohol withdrawal seizure with a concurrent alanine aminotransferase increase in the same participant (1/930; 0.1%). Neither the seizure nor the enzyme rise is attributable to vaccination, and no serious adverse event attributed to vaccination was reported in any of the ten included trials (0/919).

Five trials reported the proportion of participants with any solicited adverse event [20,21,22,23,30]; three reported the proportion experiencing one specific event such as injection-site pain or specific systemic reactions [24,25,29]; and one reported the absolute number of events not per participant [32]. However, local reactions—such as local findings at the site of injection (redness, swelling/induration, bruising or pain) or injection site pain—were frequently reported, ranging from around 30% in the South Korean trials [20,21] to up to 100% in the two earliest US phase I studies [25,29]. Injection-site pain was the most commonly reported AE in the US DNA vaccine trials [22,23,24,25,29,30], ranging from 28% in [30] to up to 96% in [24]. AEs seem to be less common among the Hantavax^®^ trials [20,21]. Systemic reactions—such as fever, fatigue, headache, myalgia, and malaise—were less frequent than local reactions, ranging from 25.2% in the South Korean phase III trial [20] to 65% in the ANDV trial [22]. The recent registry-only trial NCT03718130 [30] raised some concern on overall safety of DAN vaccines.

### 3.4. Quality Evaluation of Included Studies

Among the randomized trials, only the ANDV trial [22] was at *low risk* of bias across all five domains: double blind, placebo-controlled, followed to day 337, with exact confidence intervals reported for pre-specified outcomes at pre-specified days. Three raised *some concerns* [24,25,26]. In the 2014 trial [25], the concern is analytical: the abstract reports response rates among participants completing all three vaccinations while the Results report them among participants receiving at least two, and the denominator appearing in the text is the more favorable one. In the phase IIa trial [24], randomization, blinding and outcome measurement were appropriately reported, but the headline figure is the proportion responding at one or more of nine sampling points, a composite that is not the pre-specified primary analysis and that can only increase with the number of time points sampled. In the 2024 trial [23], five of 27 participants were excluded from the immunogenicity population after the fact on visit-window criteria, and blinding of the laboratory is not described.

The fifth randomized trial [30] was judged to be at *high risk* of bias. It was an open label with no masking, and its safety outcomes are participant-reported and unblended. One of its six arms was closed after enrolling only two participants. Moreover, the registered title and protocol specified immunogenicity alongside safety and reactogenicity as study outcomes, whereas only part of the results were made available. Notably, the study data were made available three years after the study had been completed.

The five nonrandomized studies [20,21,29,31,32] all stated a clear objective, pre-specified eligibility criteria, and antibody thresholds, and delivered the intervention consistently. Their ratings diverge on three items. Representativeness: Four enrolled screened healthy volunteers, whereas the 1999 trial [32] enrolled agricultural workers in a county with one of the highest HFRS incidences in Korea, and is the only study in this review conducted in the population for whom the vaccine is indicated. Sample size: Only the two phase III trials [20,21] pre-specified a power calculation, of 116 and 190 participants, respectively. Loss to follow-up was a point of interest: The 2016 trial [20] analyzed immunogenicity in 142 of 226 enrolled in its per-protocol, and the 1999 trial [32] tested 64 participants at one month but only 14 at thirteen months.

On this basis, the 2020 phase III trial [21] was rated *good*, the 2001, 2012 and 2016 studies [20,29,31] *fair*, and the 1999 study [32] *poor*. The 1999 rating reflects attrition above 75% by the final time point, the absence of any sample-size justification, and an internal inconsistency in the report itself, which gives 13% neutralizing seropositivity one month after the first dose in the text, while the corresponding figure reports three of 23 and is labelled 23%. Blinded outcome assessment is documented in only two of the five [27,29], both of which had sera read blind at a laboratory independent of the manufacturer. Nine of the ten trials were funded by the developer of the vaccine under study or by the military program that developed it, and in each the sponsor designed, conducted and analyzed the trial. The complete quality evaluations are reported in Tables S3 and S4.

## 4. Discussion

This systematic review assessed ten clinical randomized or nonrandomized trials evaluating various developed or under-development vaccines against hantaviruses across 961 healthy adult volunteers. Both the inactivated Hantavax^®^ vaccine and the DNA candidates induced measurable neutralizing antibody responses, but the magnitude of those responses largely varied by the adopted measure. Monovalent DNA constructs achieved the highest homologous rates, reaching 100% against HTNV and 83.3% against PUUV by PRNT_50_, whereas the bivalent HTNV/PUUV construct reached only 33.3% for both viruses [23]. For Hantavax^®^, fewer than three in five recipients showed a neutralizing response after any schedule studied, a figure essentially unchanged across the twenty-five years separating the earliest from the most recent trial [20,21,31,32].

A first major issue in interpreting these results is the marked heterogeneity across trials in terms of antigen dose, number and timing of doses, and timing of post-vaccination assessment, as well as the laboratory assays used to measure immunogenicity. Neutralizing antibody responses were evaluated using PRNT, PsVNA and IFA, each with assay-specific positivity thresholds, which makes numerical comparisons across platforms difficult. Among the available neutralization assays, PRNT_50_ is widely regarded as the gold-standard method for measuring virus-neutralizing antibodies, because it directly quantifies the reduction of plaques produced by wild-type virus infection in cell culture and has been extensively standardized and used as a benchmark in vaccine studies across different viral pathogens [33,34]. In the context of hantavirus vaccine development, PRNT_50_ was therefore adopted as the primary reference assay also in the present synthesis, as it was the only method consistently reported across all seven included trials and offers a more conservative and physiologically grounded estimate of neutralizing activity than surrogate systems [35]. Several of the more recent DNA vaccine trials complemented PRNT_50_ with pseudovirion-based neutralization assays such as PsVNA_50_, which employ vesicular stomatitis virus (VSV) or other viral backbones expressing hantavirus glycoproteins to measure the dilution of serum required to reduce pseudovirion infectivity by 50%. Across the trials that reported both PRNT_50_ and PsVNA_50_ [22,23,24], these assays tend to yield higher neutralizing titers while maintaining a reasonable correlation with live-virus neutralization. However, because they rely on engineered surrogate viral systems, their readouts are not yet fully validated as correlates of clinical protection for hantavirus infection, and higher titers in pseudovirus assays should be interpreted cautiously rather than assumed to indicate superior vaccine-induced protection [36]. Furthermore, live-virus PRNT_50_ assays require more complex Biosafety Level 3 (BSL-3) facilities, given the potential for severe disease following aerosol exposure, whereas pseudovirion-based assays can often be performed under Biosafety Level 2 (BSL-2) conditions. Thus, inevitably, this difference in safety requirements influences the choice of which neutralization methods are feasible in routine vaccine studies [36]. In addition to neutralization assays, some trials [20,21] also reported IFA titers, which measure binding rather than neutralizing antibodies and are therefore reported separately throughout this review [35].

A second important limitation is that no correlate or surrogate of protection has been validated for hantavirus infection in humans [37,38]. Antibody titers can only be interpreted as evidence of protection once such a relationship has been demonstrated, and at least three drawbacks deserve mention. First, titers obtained with different neutralization assays are not on a common scale [39], and a comparable systematic divergence has been reported for Andes virus [36]; none of the included trials reported calibration of its neutralization assay against a common reference serum. Second, binding and functional assays give discordant results, as shown in Table 2a–c. Finally, the contribution of cell-mediated immunity is incompletely characterized, and no included trial reported a cellular endpoint, although passive-transfer and monoclonal-antibody work indicates that glycoprotein-directed neutralizing antibodies are sufficient for protection in animal models [40,41]. Until these gaps are closed, a titer above a protocol-defined threshold is more a laboratory result than a statement about clinical risk [37,38].

A third, related drawback of the current research scenario lies in the absence of clinical efficacy data across the included trials: none was sufficiently powered to evaluate vaccine protection against symptomatic infection, hemorrhagic fever with renal syndrome (HFRS) or hantavirus cardiopulmonary syndrome (HCPS) in real-world conditions. Overall, these findings are based on ten studies (mostly early-phase trials) and a low number of participants, so the evidence remains preliminary. This drawback can be explained, at least partially, by the low incidence of both HFRS and HCPS and the geographic clustering and temporal unpredictability of hantavirus infections, which complicate the design and execution of classical phase III efficacy trials and may also limit the commercial attractiveness of vaccine development in this field [42,43,44]. Consistent with this, nine of the ten included trials were funded by the developer of the vaccine under study or by the military program that developed it, and in each the sponsor designed, conducted and analyzed the trial, which bears on the likelihood that unfavorable arms, time points, or assays were reported as fully as favorable ones.

This review has limitations of its own. It was not prospectively registered, and eligibility was restricted to English-language reports, which matters because inactivated hantavirus vaccines have been used at scale in China since the 1990s, and vast literature is published in Chinese [45,46]; therefore, the absence of Chinese trials should not be read as an absence of Chinese evidence. The evidence base itself is thin, with evaluable arms of two to thirty participants, follow-up of one week up to seventeen months, and no clinical endpoint; finally, only one trial enrolled the occupationally exposed population for whom vaccination would actually be considered, and no data exist for children, pregnant women or immunocompromised individuals.

## 5. Conclusions

Taken together, these issues limit the possibility of obtaining summary quantitative estimates of immunogenicity and safety, which could be potentially useful for establishing effective public health strategies. Within the available evidence, no serious safety signal was identified, but safety remains insufficiently characterized, particularly with regard to uncommon, delayed, or population-specific adverse events. Furthermore, immunogenicity varies with construct, dose, schedule and, above all, with the assay used; overall certainty was very low. Indeed, the recent Andes orthohantavirus outbreak linked to expedition cruise ship travel in South America has underscored the importance of a prompt response against hantaviruses, as well as against other potential public health threats [47,48]. No vaccine is licensed against New-World hantaviruses, and the human evidence for that target consists of a single first-in-human study, so preparedness for such events cannot currently rely on vaccination. Within this context [49], future research should aim to standardize immunogenicity endpoints and assay thresholds, establish robust correlates of protection, explore innovative trial designs adapted to low-incidence diseases, and integrate vaccination strategies into broader risk-reduction approaches, particularly for high-risk groups, such as expedition travelers, military personnel and residents of endemic rural areas.

## Figures and Tables

**Table 1:** General (**a**) and selected interventional characteristics (**b**) of the included studies.

(**a**)									
**First Author**	**Journal**	**Year**	**Country**	**Total Sample**	**Mean Age** **(SD)**	**Trial ID, Phase** **and Status**	**Blinding Design**	**Outcomes**	**Funding**
Cho [32]	Vaccine	1999	S. Korea	64	NR	NR	Open-Nonrandom	I; S	NR
Sohn [31]	Yonsei Med J	2001	S. Korea	30	NR	NR	Open-Nonrandom	I;	NR
Boudreau [29]	Vaccine	2012	US	28	32.0 (IQR 19-47)	Phase I; NR	Open-Nonrandom	I; S	US Army MRDC
Hooper [25]	Clin Microbial Infect	2014	US	27	NR	Phase I; NCT01502345	Open-Random	I; S	US Army MRDC
Song [20]	Vaccine	2016	S. Korea	226	43.3 (14.1)	Phase III; NCT02360514	Open- Nonrandom	I; S	Green Cross Corporation
Hooper [24]	Vaccines	2020	US	130	NR	Phase IIa; NCT02116205	Double-Random	I; S	US Army MRDC
Song [21]	Vaccine	2020	S. Korea	320	41.1 (9.4)	Phase III; NCT02553837	Open-Nonrandom	I; S	Green Cross Corporation
Paulsen [22]	J Infect Dis	2023	US	48	34.7 (8.6)	Phase I; NCT03682107	Double-Random	I; S	NIAID
Hooper [23]	Npj Vaccines	2024	US	27	NR	Phase I; NCT02776761	Single-Random	I; S	US Army MRDC
NCT03718130 [30]	ClinicalTrials.gov	2025	US	61	NR	Phase I; NCT03718130	Open-Random	I; S	US Army MRDC
(**b**)									
**First Author**	**Year**	**Allocation**	**Tested Vaccine(s)** **and Number of Doses**			**Vaccine Dose and Schedule (Days)**			**Delivery** **Methods**
Cho [32]	1999	Single arm	Hantavax^®^; 2 doses+booster			**Arm1:** 0.5 mL d1, d29 + d365 (booster) (administered volume)			Classic IM
Sohn [31]	2001	Single arm	Hantavax^®^; 2 doses			**Arm1:** 0.5 mL d1, d29 (administered volume)			Classic IM
Boudreau [29]	2012	Multi-arm, 3 arms	Monovalent/bivalent DNA plasmid; 3 doses			**Arm1:** HTNV 8 μg DNA/4 mg gold d0, d28, d56 **Arm2:** PUUV 8 μg DNA/4 mg gold d0, d28, d56 **Arm3:** HTNV 4 μg DNA/2 mg gold + PUUV 4 μg DNA/2 mg gold (8 μg DNA/4 mg gold) d0, d28, d56			Classic IM
Hooper [25]	2014	Multi-arm, 3 arms	Monovalent/bivalent DNA plasmid; 3 doses			**Arm1:** HTNV 2 mg d1, d28, d56 **Arm2:** PUUV 2 mg d1, d28, d56 **Arm3:** HTNV 1 mg + PUUV 1 mg (2 mg total DNA vaccines) d1, d28, d56			TriGrid
Song [20]	2016	Single arm	Hantavax^®^; 2 doses+booster			**Arm1:** 0.5 mL d1, d29 + d365 (booster) (administered volume)			Classic IM
Hooper [24]	2020	Multi-arm, 4 arms, placebo dose in arms 2 and 4	Bivalent DNA plasmid; 2/3 doses + booster ^¥^			**Arm1:** HTNV 0.5 mg + PUUV 0.5 mg d0, d28, d56 + d168 (booster) **Arm2:** HTNV 0.5 mg + PUUV 0.5 mg d0, d28, d56(Plac) + d168 (booster) **Arm3:** HTNV 1 mg + PUUV 1 mg d0, d28, d56 + d168 (booster) **Arm4:** HTNV 1 mg + PUUV 1 mg (2 mg total DNA vaccines) d0, d28, d56(Plac) + d168 (booster)			TriGrid
Song [21]	2020	Single arm	Hantavax^®^; 3 doses + booster			**Arm1:** 0.5 mL d1, d29, d57 + d394 (booster) (administered volume)			Classic IM
Paulsen [22]	2023	Multi-arm, 4 arms, placebo dose in arms 1 and 3	Monovalent ANDV DNA plasmid 2/3 doses+booster ^¥^			**Arm1:** ANDV 2 mg d1, d29, d57 (Plac) + d169 (booster) **Arm2:** ANDV 2 mg d1, d29, d57 + d169 (booster) **Arm3:** ANDV 4 mg d1, d29, d57 (Plac) + d169 (booster) **Arm4:** ANDV 4 mg d1, d29, d57 + d169 (booster)			PharmaJet Stratis
Hooper [23]	2024	Multi-arm, 3 arms, no placebo	Monovalent/bivalent DNA plasmid; 3 doses+booster			**Arm1:** HTNV 2 mg d1, d29, d57 + d169 (booster) **Arm2:** PUUV 2 mg d1, d29, d57 + d169 (booster) **Arm3:** HTNV 1 mg + PUUV 1 mg (2 mg total DNA vaccines) d1, d29, d57 + d169 (booster)			PharmaJet Stratis
NCT03718130 [30]	2022	Multi-arm, 6 arms, no placebo	Monovalent/bivalent DNA plasmid; 3 doses			**Arm 1:** HTNV 0.6 mg d0, d28, d56 ID **Arm 2:** HTNV 3.0 mgd0, d28, d56 IM **Arm 3:** PUUV 0.6 mgd0, d28, d56 ID	**Arm 4:** PUUV 3.0 mg d0, d28, d56 IM **Arm 5:** HTNV 0.6 mg + PUUV 0.6 mg (1.2 mg total DNA vaccines) d0, d28, d56 ID **Arm 6:** HTNV 3 mg + PUUV 3 mg (6 mg total DNA vaccines) d0, d28, d56 IM		TriGrid

SD: standard deviation; IQR: interquartile range; NR: not reported; I: immunogenicity; S: short-term safety; Open: open label; Single: single blinding; Double: double blinding; Nonrandom: nonrandomized (single-arm trial); Random: randomized; Plac: placebo; MRDC: Medical Research and Development Command; NIAID: National Institute of Allergy and Infectious Diseases; HTNV: *Hantaan* virus DNA vaccine; PUUV: *Puumala* virus DNA vaccine; ANDV: *Andes* virus DNA vaccine; Classic IM: intramuscular, needle and syringe; ID; intradermal delivery; TriGrid: intramuscular or intradermal electroporation; PharmaJet Stratis: needle-free jet injection. ^¥^ In two arms, one of the three doses in the primary vaccination course was placebo; thus, the corresponding schedule was composed by 2 vaccine administrations + one placebo administration + one vaccine booster administration.

**Table 2:** (**a**) Immunogenicity of the inactivated whole-virus vaccine by outcome, endpoint and assay. (**b**) Seropositivity after DNA vaccine injection by included study. (**c**) Seroconversion after DNA vaccine injection by included study.

(**a**)				
**First Author**	**Primary Immunogenicity** **Outcomes and Endpoints**	**Arm(s)**	**Seropositivity Rate**	
			**PRNT_50_, n/N**	**IFA, n/N**
Cho [32]	% of subjects achieving seropositivity, expressed through PRNT_50_ antibody titers of ≥1:10 ^A^	**Arm 1:**	50.0%; 7/14	93.6%; 15/16
Song [21]	% of subjects achieving seropositivity, expressed through PRNT_50_ and IFA; PRNT_50_ antibody titers of ≥1:10 or IFA antibody titers of ≥1:32 were regarded as positive ^A^	**Arm 1:**	45.1%; 64/142	87.3%; 124/142
Song [21]	% of subjects achieving seropositivity, expressed through PRNT_50_ and IFA; PRNT_50_ antibody titers of ≥1:10 or IFA antibody titers of ≥1:32 were regarded as positive ^A^	**Arm 1:**	55.7%; 161/289	95.7%; 266/278
**First Author**	**Primary Immunogenicity** **Outcomes and Endpoints**	**Arm(s)**	**Seroconversion Rate**	
			**PRNT_50_, n/N**	**IFA, n/N**
Sohn [31]	% of subjects achieving seroconversion, expressed through PRNT_50_; PRNT_50_ antibody titers of ≥1:10 ^B^	**Arm 1**	33.3%; 10/30	NR
(**b**)				
**First Author**	**Primary Immunogenicity** **Outcomes and Endpoints**	**Arm(s)**	**Seropositivity Rates**	
			**PRNT_50_ (n/N)**	**PsVNA_50_ (n/N)**
Boudreau [29]	% of subjects achieving seropositivity, expressed through PRNT_50_ antibody titer ≥ 1:20 ^C^; see Table 1b for details by treatment arm	**Arm 1:**	30.0% ^(HTNV)^ (3/10)	NR
		**Arm 2:**	44.4% ^(PUUV)^ (4/9)	
		**Arm 3:**	44.4% ^(HTNV)^ (4/9); 33.3% ^(PUUV)^ (3/9); 22.2% ^(HTNV and PUUV)^ (2/9)	
Hooper [25]	% of subjects achieving seropositivity, expressed through PRNT_50_ antibody titer ≥ 1:20 ^C^; see Table 1b for details by treatment arm	**Arm 1:**	63.6% ^(HTNV)^ (7/11)	NR
		**Arm 2:**	75.0% ^(PUUV)^ (6/8)	
		**Arm 3:**	33.3% ^(HTNV)^ (3/9); 77.8% ^(PUUV)^; (7/9); NR ^(HTNV and PUUV)^	
Hooper [24]	% of subjects achieving seropositivity, expressed through dual PsVNA_50_ ^D^; see Table 1b for details by treatment arm	**Arm 1:**	NR	76.7% ^(HTNV and PUUV)^ (23/30)
		**Arm 2:**		73.3% ^(HTNV and PUUV)^ (22/30)
		**Arm 3:**		80.0% ^(HTNV and PUUV)^ (24/30)
		**Arm 4:**		66.7% ^(HTNV and PUUV)^ (20/30)
Paulsen [22]	% of subjects achieving seropositivity, expressed through PsVNA_50_ antibody titer ≥ 1:20 ^D^; see Table 1b for details by treatment arm	**Arm 1:**	NR	55.6%^(ANDV)^ (5/9)
		**Arm 2:**		100.0%^(ANDV)^ (9/9)
		**Arm 3:**		88.9%^(ANDV)^ (8/9)
		**Arm 4**:		88.9%^(ANDV)^ (8/9)
Hooper [23]	% of subjects achieving seropositivity, expressed through PsVNA_50_ or PRNT_50_ antibody titer ≥ 1:20 ^D^; see Table 1b for details by treatment arm	**Arm 1:**	100.0% ^(HTNV)^ (7/7); 42.9% ^(PUUV)^ (3/7)	100.0% ^(HTNV)^ (7/7); 100.0% ^(PUUV)^ (7/7)
		**Arm 2:**	33.3% ^(HTNV)^ (2/6); 83.3% ^(PUUV)^; (5/6)	50.0% ^(HTNV)^ (3/6); 100.0% ^(PUUV)^ (6/6)
		**Arm 3:**	44.4% ^(HTNV)^ (4/9); 77.8% ^(PUUV)^ (7/9)	77.8% ^(HTNV)^ (7/9); 88.9% ^(PUUV)^ (8/9)
NCT03718130 [30]	% of subjects achieving seropositivity, expressed through PsVNA_50_ antibody titer ≥ 1:20 ^C^; see Table 1b for details by treatment arm	**Arm 1:**	NR	100.0% ^(HTNV)^ (4/4)
		**Arm 2:**		75% ^(HTNV)^ (9/12)
		**Arm 3:**		75% ^(PUUV)^ (3/4)
		**Arm 4:**		33% ^(PUUV)^ (4/12)
		**Arm 5:**		0% ^(HTNV+PUUV)^ (0/2)
		**Arm 6:**		50% ^(HTNV+PUUV)^ (1/2)
(**c**)				
**First Author**	**Primary Immunogenicity** **Outcomes and Endpoints**	**Arm(s)**	**Seroconversion Rates**	
			**PRNT_50_**	**PsVNA_50_**
Paulsen [22]	% of subjects achieving seroconversion, defined as a post-vaccination ANDV-specific titer through PsVNA_50_ of ≥40 if baseline titer < 20, or a minimum 4-fold rise if baseline titer ≥ 20 ^E^; see Table 1b for details by treatment arm	**Arm 1:**	NR	55%^(ANDV)^ (n/N NR)
		**Arm 2:**		88%^(ANDV)^ (n/N NR)
		**Arm 3:**		60%^(ANDV)^ (n/N NR)
		**Arm 4:**		89%^(ANDV)^ (n/N NR)
Hooper [23]	% of subjects achieving seroconversion, defined as a post-vaccination single or combined HTNV or PUUV specific titer through PsVNA_50_ or PRNT_50_ of ≥40 or 4-fold rise over baseline ^D^; see Table 1b for details by treatment arm	**Arm 1:**	100.0% ^(HTNV)^ (7/7); 42.9% ^(PUUV)^ (3/7); 42.9% ^(HTNV and PUUV)^ (3/7)	100.0% ^(HTNV)^ (7/7); 71.4% ^(PUUV)^ (5/7); 57.1% ^(HTNV and PUUV)^ (4/7)
		**Arm 2:**	50.0% ^(HTNV)^ (3/6); 83.3% ^(PUUV)^ (5/6); 33.3% ^(HTNV and PUUV)^ (2/6)	50.0% ^(HTNV)^ (3/6); 100% ^(PUUV)^ (6/6); 66.7% ^(HTNV and PUUV)^ (4/6)
		**Arm 3:**	33.3% ^(HTNV)^ (7/9); 77.8% ^(PUUV)^ (7/9); 33.3% ^(HTNV and PUUV)^ (3/9)	77.8% ^(HTNV)^ (6/9); 88.9% ^(PUUV)^ (8/9); 77.8% ^(HTNV and PUUV)^ (7/9)
NCT03718130 [30]	% of subjects achieving seroconversion, defined as a post-vaccination single or combined HTNV or PUUV specific titer through PsVNA_50_ of ≥40 or 4-fold rise over baseline ^C^; see Table 1b for details by treatment arm	**Arm 1:**	NR	100.0% ^(HTNV)^ (4/4)
		**Arm 2:**		50% ^(HTNV)^ (6/12)
		**Arm 3:**		75% ^(PUUV)^ (3/4)
		**Arm 4:**		75% ^(PUUV)^ (9/12)
		**Arm 5:**		0% ^(HTNV + PUUV)^ (0/2)
		**Arm 6:**		50% ^(HTNV+PUUV)^ (1/2)

PRNT_50_: plaque reduction neutralization antibody test; IFA: immunofluorescence antibody assay. PRNT_50_: highest serum dilution capable of reducing the standard viral dose by 50%; PsVNA_50_: pseudovirion neutralization assay dilution capable of reducing viral activity by 50%; HTNV: *Hantaan* virus; PUUV: *Puumala* virus; ANDV: *Andes* virus; NR: not reported. ^A^ Immunogenicity outcomes extracted at 13 month (1 month after booster dose); ^B^ Immunogenicity outcomes extracted at d56 (1 month after second or third dose, depending on schedule; ^C^ Immunogenicity outcomes extracted at d84 (1 month after second or third dose, depending on schedule); ^D^ Immunogenicity outcomes extracted at d196 (1 month after a primary or booster dose); ^E^ Immunogenicity outcomes extracted at d337 (6 months after a primary or booster dose).

**Table 3:** Short-term safety outcomes, with exact 95% confidence intervals, in the safety population.

First Author	Primary Safety Outcomes and Endpoints	Follow-Up	Rates of AEs		Rates of SAEs
			Local Reactions	Systemic Reactions	
Cho [32]	Time-specific and cumulative rates of SAEs and AEs—local and systemic reactions—14 days after each injection	1 week after last dose	Reported as events, not per participant	Reported as events, not per participant	0/64 (0.0%)
Boudreau [29]	Time-specific and cumulative rates of SAEs and AEs—local and systemic reactions—14 days after each injection	1 month after last dose	Skin discoloration after 100% of doses; injection-site pain after 48–56% of doses	Fatigue 10/28 (35.7%); malaise 7/28 (25.0%); lymphadenopathy 6/28 (21.4%)	0/27 (0%)
Hooper [25]	Unsolicited SAEs and AEs, 14 days after each injection	6 months after last dose	Injection-site pain: 28/31 (90.3%)	Headache 8/31 (25.8%); myalgia 7/31 (22.6%); fatigue 6/31 (19.4%)	0/27 (0%)
Song [20]	Time-specific and cumulative rates of SAEs and AEs—local and systemic reactions—7 days after each injection	6 months after last dose	Any local reactions: 108/226 (47.8%)	Any systemic reactions: 57/226 (25.2%)	0/226 (0%)
Hooper [25]	Unsolicited SAEs and AEs, 14 days after each injection	12 months after last dose	Injection-site pain: 125/130 (96.2%)	Fatigue 38/130 (29.2%); headache 31/130 (23.8%); myalgia 23/130 (17.7%)	0/130 (0%)
Song [21]	Time-specific and cumulative rates of SAEs and AEs—local and systemic reactions—7 days after each injection	4 months after last dose	Any local reactions: 113/309 (37%)	Any systemic reactions: 66/309 (21.4%)	0/309 (0%)
Paulsen [22]	Time-specific and cumulative rates of SAEs and AEs—local and systemic reactions—7 days after each injection	6 months after last dose	Any local reactions: 47/48 (97.9%)	Any systemic reactions: 31/48 (64.6%)	0/48 (0%)
Hooper [23]	Time-specific and cumulative rates of SAEs and AEs—local and systemic reactions—14 days after each injection	6 months after last dose	Any local reactions: 23/27 (85.2%) Injection-site pain: 21/27 (77.8%);	Headache 13/27 (48.1%); fatigue 10/27 (37.0%)	0/27 (0%)
NCT03718130 [30]	Time-specific and cumulative rates of SAEs and AEs—local and systemic reactions—14 days after each injection	6 months after last dose	Any local reactions: 10/36 (28%)	Any systemic reactions: 1/36 (3%)	1/61 (1%)

AEs: adverse events; SAEs: serious adverse events; NR: not reported.

## Data Availability

All data underlying this review are derived from published studies and are reported in full within the manuscript and its Supplementary Materials.

## Supplementary Materials

The following supporting information can be downloaded at https://www.mdpi.com/article/10.3390/vaccines14100898/s1, Table S1: List of the full electronic search strategies used across all consulted databases (PubMed, EMBASE, Scopus); Table S2: List of the 9 excluded studies after full text screening, and reasons for exclusion; Table S3: Risk of bias assessment of the included randomized studies using the revised Cochrane risk-of-bias tool for randomized trials; Table S4: Risk of bias assessment of the included nonrandomized studies using the NIH Quality Assessment Tool for Before–After [Pre–Post] Studies with No Control Group; Table S5: PRISMA 2020 checklist.

## Abbreviations

The following abbreviations are used in this manuscript:

AEs	Adverse Events
ANDV	Andes Virus
BSL	BioSafety Level
DNA	DeoxyriboNucleic Acid
HCPS	Hantavirus Cardio-Pulmonary Syndrome
HFRS	Haemorrhagic Fever with Renal Syndrome
HTNV	Hantaan Virus
IFA	ImmunoFluorescence Antibody Assay
MV	Motor Vessel
PRISMA	Preferred Reporting Items for Systematic Reviews and Meta-Analyses
PRNT	Plaque Reduction Neutralization Antibody Test
PsVNA	PseudoVirion Neutralization Assay
PUUV	Puumala Virus
RoB 2	Risk-of-Bias Tool
RNA	RiboNucleic Acid
SAEs	Serious Adverse Events
