# Supplementary material for: Vaccines Against Hantavirus Infections: A Systematic Literature Review
Source: Vaccines (Basel). 2026 Oct 13;14(10):898. doi: 10.3390/vaccines14100898 (PMC13709835; doi:10.3390/vaccines14100898)
Supplement: Supplementary file 1 [file vaccines-14-00898-s001.zip › vaccines-4574357-supplementary.pdf]

Supplementary Material

Table S1. Full adopted search strategy for each database.

Databases	Search string
- **PubMed**	836 results on 23.9.2026<br><br>("Orthohantavirus"[MeSH] OR "Hantavirus Infections"[MeSH] OR hantavirus*[tiab] OR orthohantavirus*[tiab] OR hantaan[tiab] OR puumala[tiab] OR "sin nombre"[tiab] OR andes[tiab] OR dobrava[tiab] OR seoul virus[tiab] OR HFRS[tiab] OR HCPS[tiab] OR HPS[tiab] OR "hantavirus cardiopulmonary syndrome"[tiab] OR "hemorrhagic fever with renal syndrome"[tiab] OR "epidemic haemorrhagic fever"[tiab] OR "epidemic hemorrhagic fever"[tiab] OR "nephropathia epidemica"[tiab]) AND ("Vaccines"[MeSH] OR "Vaccination"[MeSH] OR "Immunization"[MeSH] OR vaccin*[tiab] OR immunis*[tiab] OR immuniz*[tiab] OR hantavax[tiab] OR "inactivated vaccine"[tiab] OR "DNA vaccine"[tiab] OR plasmid[tiab] OR bivalent[tiab]) AND humans[MeSH]
- **EMBASE**	1365 results on 23.9.2026<br><br>('hantavirus'/exp OR 'hantavirus infection'/exp OR hantavirus*:ti,ab OR orthohantavirus*:ti,ab OR hantaan:ti,ab OR puumala:ti,ab OR 'sin nombre':ti,ab OR andes:ti,ab OR dobrava:ti,ab OR 'seoul virus':ti,ab OR hfrs:ti,ab OR hcps:ti,ab OR hps:ti,ab OR 'haemorrhagic fever with renal syndrome':ti,ab OR ‘Hantavirus cardiopulmonary syndrome’:ti,ab OR 'epidemic haemorrhagic fever':ti,ab OR 'epidemic hemorrhagic fever':ti,ab OR 'nephropathia epidemica':ti,ab) AND ('vaccine'/exp OR 'vaccination'/exp OR 'immunization'/exp OR vaccin*:ti,ab OR immunis*:ti,ab OR immuniz*:ti,ab OR hantavax:ti,ab OR 'inactivated vaccine':ti,ab OR 'dna vaccine':ti,ab OR plasmid:ti,ab OR bivalent:ti,ab) AND [humans]/lim
- **Scopus**	780 results 23.9.2026<br><br>TITLE-ABS-KEY (hantavirus* OR orthohantavirus* OR hantaan OR puumala OR hantavirus infect* OR HFRS OR HCPS OR HPS OR haemorrhagic fever with renal syndrome OR Hantavirus cardiopulmonary syndrome) AND (vaccin* OR immunis* OR immuniz* OR hantavax OR inactivated vaccine OR DNA vaccine OR plasmid OR bivalent) AND (human* OR patient* OR volunteer* OR participant*)

Table S2. Reason(s) for exclusion.

First author, year	Trial ID	Reason(s) for exclusion
Zhu 1994 (1)		Different study design
Gong 2000 (2)	11860948	Chinese language
McCain 2000 (3)		Different study design
Ruan 2001 (4)	11986705	Chinese language
NCT04323904 (5)		Results not available and different study design—under development
NCT06859619 (6)		Results not available and different study design—under development
Zheng 2018 (7)		PRNT50, PSvNA50 or IFA not assessed for immunogenicity evaluation
Khan 2019 (8)		Different study design
NCT04333459 (9)		Results not available—completed

Table S3. Risk of bias of the included randomized studies assessed using the revised Cochrane risk-of-bias tool for randomized trials.

Study ID	Randomization process	Deviations from intended interventions	Missing outcome data	Measurement of the outcome	Selection of reported results	Overall bias
Hooper 2014 (10)	Some concerns	Some concerns	Some concerns	Low risk	Some concerns	Some concerns
Hooper 2020 (11)	Low risk	Low risk	Low risk	Low risk	Some concerns	Some concerns
Paulsen 2023 (12)	Low risk	Low risk	Low risk	Low risk	Low risk	Low risk
Hooper (13)	Low risk	Some concerns	Some concerns	Some concerns	Some concerns	Some concerns
NCT03718130 (14)<br>Results posting only	Some concerns	Some concerns	Some concerns	High risk	High risk	High risk

Table S4. Methodological quality of the five nonrandomized studies (NIH Quality Assessment Tool for Before–After [Pre–Post] Studies With No Control Group).

NIH item	Cho 1999 (18)	Sohn 2001 (19)	Boudreau 2012 (15)	Song 2016 (16)	Song 2020 (17)
1. Was the study question or objective clearly stated?	Yes	Yes	Yes	Yes	Yes
2. Were eligibility and selection criteria pre-specified and clearly described?	Yes	Yes	Yes	Yes	Yes
3. Were participants representative of those who would be eligible for the intervention in the population of interest?	Yes—agricultural workers in a high-incidence endemic county	No—screened healthy volunteers	No—screened healthy volunteers	No—screened healthy volunteers	No—screened healthy volunteers
4. Were all eligible participants meeting the pre-specified criteria enrolled?	Cannot determine	Cannot determine	Yes	Cannot determine—30 of 256 volunteers excluded at screening	Cannot determine
5. Was the sample size sufficiently large to provide confidence in the findings?	No—no power calculation; 14 participants at the final time point	No—30 participants; no power calculation	No—10, 9 and 9 per group	Yes—116 and 80 pre-specified for the primary and secondary objectives	Yes— 90 pre-specified for the primary objective
6. Was the intervention clearly described and delivered consistently?	Yes	Yes	Yes	Yes	Yes
7. Were outcome measures pre-specified, clearly defined, valid, reliable and assessed consistently?	Yes—PRNT, IFA and ELISA with stated thresholds	Yes—PRNT with and without complement, HAI, ELISA, and HDPA	Yes—PRNT50 ≥20	Yes—PRNT50 ≥1:10; IFA ≥1:32	Yes—PRNT50 ≥1:5 and ≥1:10; IFA ≥1:32
8. Were outcome assessors blinded to the intervention received?	Not reported	Yes—read at USAMRIID using a laboratory blinding method	Yes—blinded samples screened and retested	Not reported	Not reported
9. Was loss to follow-up after baseline 20% or less, and were those lost accounted for?	No—64 tested at one month, 24 at twelve months, and 14–16 at thirteen months	Yes—90 sera from 30 participants; complete	Yes—27 of 28 completed	No—142 of 226 in the per-protocol set, and 64 at the later time points	Yes—289 of 320 in the per-protocol set, with reasons stated
10. Did the statistical methods examine change in outcome from before to after, with p-values reported?	Partly—pre- and post-vaccination titers compared descriptively; no p-values	Yes—correlation between assays tested ($r = 0.58$, $p < 0.01$)	Partly—descriptive, with binomial proportions and exact 95% CIs	Yes—Kaplan–Meier for duration; t-test and chi-square for comparisons	Yes—McNemar and Cochran Q for change across vaccinations
11. Were outcome measures taken multiple times before and after the intervention?	Yes—days 0, 30, 60, 390	Yes—before and 4 weeks after each of two doses	Yes—days 0, 28, 56, 84, 140, 180	Yes—months 0, 2, 13, 14, 25, 37, 49	Yes—months 0, 3, 13, 14, 15, 17
12. If the intervention was delivered at the group level, did the analysis account for individual-level data?	Not applicable	Not applicable	Not applicable	Not applicable	Not applicable
Overall quality rating	Poor	Fair	Fair	Fair	Good

Ratings: Yes / No / Partly / Cannot determine / Not reported / Not applicable. The 1999 study is rated poor because of attrition above 75% by the final time point, the absence of a sample-size justification, and an internal inconsistency in the report, which gives 13% neutralizing seropositivity one month after the first dose in the text while the corresponding figure shows 3 of 23 and is labelled 23%. The 2012 trial allocated participants to three groups by sequential enrolment rather than randomization; this tool appraises its within-group pre–post comparisons only, and its between-group comparisons are not used in this review. The 2001 and 2012 studies are the only two in which blinded outcome assessment is documented.

Table S5. PRISMA 2020 checklist..

Section and Topic	Item #	Checklist item	Location where item is reported
**TITLE**			
Title	1	Identify the report as a systematic review.	Lines 2-3
**ABSTRACT**			
Abstract	2	See the PRISMA 2020 for Abstracts checklist.	Lines 12-34
**INTRODUCTION**			
Rationale	3	Describe the rationale for the review in the context of existing knowledge.	Lines 39-75
Objectives	4	Provide an explicit statement of the objective(s) or question(s) the review addresses.	Lines 76-88
**METHODS**			
Eligibility criteria	5	Specify the inclusion and exclusion criteria for the review and how studies were grouped for the syntheses.	Lines 93-158
Information sources	6	Specify all databases, registers, websites, organisations, reference lists and other sources searched or consulted to identify studies. Specify the date when each source was last searched or consulted.	Lines 159-160
Search strategy	7	Present the full search strategies for all databases, registers and websites, including any filters and limits used.	Lines 159-165 and Table S1
Selection process	8	Specify the methods used to decide whether a study met the inclusion criteria of the review, including how many reviewers screened each record and each report retrieved, whether they worked independently, and if applicable, details of automation tools used in the process.	Lines 166-169
Data collection process	9	Specify the methods used to collect data from reports, including how many reviewers collected data from each report, whether they worked independently, any processes for obtaining or confirming data from study investigators, and if applicable, details of automation tools used in the process.	Lines 158-159
Data items	10a	List and define all outcomes for which data were sought. Specify whether all results that were compatible with each outcome domain in each study were sought (e.g. for all measures, time points, analyses), and if not, the methods used to decide which results to collect.	Lines 178-269
	10b	List and define all other variables for which data were sought (e.g. participant and intervention characteristics, funding sources). Describe any assumptions made about any missing or unclear information.	Table 1
Study risk of bias assessment	11	Specify the methods used to assess risk of bias in the included studies, including details of the tool(s) used, how many reviewers assessed each study and whether they worked independently, and if applicable, details of automation tools used in the process.	Lines 169-176
Effect measures	12	Specify for each outcome the effect measure(s) (e.g. risk ratio, mean difference) used in the synthesis or presentation of results.	Not applicable
Synthesis methods	13a	Describe the processes used to decide which studies were eligible for each synthesis (e.g. tabulating the study intervention characteristics and comparing against the planned groups for each synthesis (item #5)).	Table 1
	13b	Describe any methods required to prepare the data for presentation or synthesis, such as handling of missing summary statistics, or data conversions.	Not applicable

Section and Topic	Item #	Checklist item	Location where item is reported
	13c	Describe any methods used to tabulate or visually display results of individual studies and syntheses.	Not applicable
	13d	Describe any methods used to synthesize results and provide a rationale for the choice(s). If meta-analysis was performed, describe the model(s), method(s) to identify the presence and extent of statistical heterogeneity, and software package(s) used.	Not applicable
	13e	Describe any methods used to explore possible causes of heterogeneity among study results (e.g. subgroup analysis, meta-regression).	Not applicable
	13f	Describe any sensitivity analyses conducted to assess robustness of the synthesized results.	Not applicable
Reporting bias assessment	14	Describe any methods used to assess risk of bias due to missing results in a synthesis (arising from reporting biases).	Not applicable
Certainty assessment	15	Describe any methods used to assess certainty (or confidence) in the body of evidence for an outcome.	Not applicable
**RESULTS**			
Study selection	16a	Describe the results of the search and selection process, from the number of records identified in the search to the number of studies included in the review, ideally using a flow diagram.	Lines 272-284 and figure 1
	16b	Cite studies that might appear to meet the inclusion criteria, but which were excluded, and explain why they were excluded.	Table S2
Study characteristics	17	Cite each included study and present its characteristics.	Table 1
Risk of bias in studies	18	Present assessments of risk of bias for each included study.	Supplementary table S3-S4
Results of individual studies	19	For all outcomes, present, for each study: (a) summary statistics for each group (where appropriate) and (b) an effect estimate and its precision (e.g. confidence/credible interval), ideally using structured tables or plots.	Tables 2a, 2b, 2c
Results of syntheses	20a	For each synthesis, briefly summarise the characteristics and risk of bias among contributing studies.	Lines 766-821
	20b	Present results of all statistical syntheses conducted. If meta-analysis was done, present for each the summary estimate and its precision (e.g. confidence/credible interval) and measures of statistical heterogeneity. If comparing groups, describe the direction of the effect.	Not applicable
	20c	Present results of all investigations of possible causes of heterogeneity among study results.	Not applicable
	20d	Present results of all sensitivity analyses conducted to assess the robustness of the synthesized results.	Not applicable
Reporting biases	21	Present assessments of risk of bias due to missing results (arising from reporting biases) for each synthesis assessed.	Not applicable
Certainty of evidence	22	Present assessments of certainty (or confidence) in the body of evidence for each outcome assessed.	Not applicable
**DISCUSSION**			
Discussion	23a	Provide a general interpretation of the results in the context of other evidence.	Lines 823-895
	23b	Discuss any limitations of the evidence included in the review.	Lines 900-904

Section and Topic	Item #	Checklist item	Location where item is reported
	23c	Discuss any limitations of the review processes used.	Lines 896-900
	23d	Discuss implications of the results for practice, policy, and future research.	Lines 906-932
**OTHER INFORMATION**			
Registration and protocol	24a	Provide registration information for the review, including register name and registration number, or state that the review was not registered.	Not provided
	24b	Indicate where the review protocol can be accessed, or state that a protocol was not prepared.	Not indicated
	24c	Describe and explain any amendments to information provided at registration or in the protocol.	Not applicable
Support	25	Describe sources of financial or non-financial support for the review, and the role of the funders or sponsors in the review.	Line 945
Competing interests	26	Declare any competing interests of review authors.	Line 952
Availability of data, code and other materials	27	Report which of the following are publicly available and where they can be found: template data collection forms; data extracted from included studies; data used for all analyses; analytic code; any other materials used in the review.	Lines 950-952

From: Page MJ, McKenzie JE, Bossuyt PM, Boutron I, Hoffmann TC, Mulrow CD, et al. The PRISMA 2020 statement: an updated guideline for reporting systematic reviews. BMJ 2021;372:n71. doi: 10.1136/bmj.n71. This work is licensed under CC BY 4.0. To view a copy of this license, visit https://creativecommons.org/licenses/by/4.0/

References

1. Zhu ZY, Tang HY, Li YJ, Weng JQ, Yu YX, Zeng RF. Investigation on inactivated epidemic hemorrhagic fever tissue culture vaccine in humans. Chin Med J (Engl). 1994 Mar;107(3):167-70.
2. Gong Z, Weng J, Zhao Z, Lan J, Chen Y, Lin C, et al. [The changes in serum antibody level after immunization with HFRS vaccine]. Zhonghua Yu Fang Yi Xue Za Zhi. 2000 Nov;34(6):351-3.
3. McClain DJ, Summers PL, Harrison SA, Schmaljohn AL, Schmaljohn CS. Clinical evaluation of a vaccinia-vectored Hantaan virus vaccine. J Med Virol. 2000 Jan;60(1):77-85.
4. Ruan Y, Liu W, Xu X, Weng SQ, Shao SK, Zhou WQ, et al. [Comparative study on antibody levels of vaccination group and control group after 4 years of immunized with type B inactivated vaccine against hemorrhagic fever with renal syndrome]. Zhonghua Shi Yan He Lin Chuang Bing Du Xue Za Zhi. 2001 Sep;15(3):274-6.
5. University of Cologne NCT04323904. Hantavirus Registry - HantaReg (HantaReg). 2025; Available from: https://clinicaltrials.gov/study/NCT04323904?cond=NCT04323904&viewType=Card&rank=1.
6. University Hospital Montpellier NCT06859619. Serological Measurement of Montpellier Professionals' Contacts with Infectious Agents Responsible for Animal-borne Diseases (SEZAM). 2025; Available from: https://clinicaltrials.gov/study/NCT06859619?viewType=Card&cond=NCT06859619&rank=1.
7. Zheng Y, Zhou BY, Wei J, Xu Y, Dong JH, Guan LY, et al. Persistence of immune responses to vaccine against haemorrhagic fever with renal syndrome in healthy adults aged 16-60 years: results from an open-label2-year follow-up study. Infect Dis (Lond). 2018 Jan;50(1):21-6.
8. Khan A, Shin OS, Na J, Kim JK, Seong RK, Park MS, et al. A Systems Vaccinology Approach Reveals the Mechanisms of Immunogenic Responses to Hantavax Vaccination in Humans. Sci Rep. 2019 Mar 18;9(1):4760.
9. U.S. Army Medical Research and Development Command NCT04333459. Safety/ Immunogenicity Hantaan Virus DNA Vaccine and Puumala Virus DNA Vaccine, For Prevention of HFRS. 2026; Available from: https://clinicaltrials.gov/study/NCT04333459?viewType=Card&cond=NCT04333459&rank=1&tab=study.
10. Hooper JW, Moon JE, Paolino KM, Newcomer R, McLain DE, Josleyn M, et al. A Phase 1 clinical trial of Hantaan virus and Puumala virus M-segment DNA vaccines for haemorrhagic fever with renal syndrome delivered by intramuscular electroporation. Clin Microbiol Infect. 2014 May;20 Suppl 5:110-7.
11. Hooper J, Paolino KM, Mills K, Kwilas S, Josleyn M, Cohen M, et al. A Phase 2a Randomized, Double-Blind, Dose-Optimizing Study to Evaluate the Immunogenicity and Safety of a Bivalent DNA Vaccine for Hemorrhagic Fever with Renal Syndrome Delivered by Intramuscular Electroporation. Vaccines (Basel). 2020 Jul 11;8(3).
12. Paulsen GC, Frenck R, Jr, Tomashek KM, Alarcon RM, Hensel E, Lowe A, et al. Safety and Immunogenicity of an Andes Virus DNA Vaccine by Needle-Free Injection: A Randomized, Controlled Phase 1 Study. The Journal of Infectious Diseases. 2024;229(1):30-8.
13. Hooper JW, Kwilas SA, Josleyn M, Norris S, Hutter JN, Hamer M, et al. Phase 1 clinical trial of Hantaan and Puumala virus DNA vaccines delivered by needle-free injection. NPJ Vaccines. 2024 Nov 17;9(1):221.

14. US. Army NCT03718130 Combination HTNV and PUUV DNA Vaccine. 2025; Available from: https://clinicaltrials.gov/study/NCT03718130?cond=NCT03718130&viewType=Card&rank=1&tab=results#results-overview.
15. Boudreau EF, Josleyn M, Ullman D, Fisher D, Dalrymple L, Sellers-Myers K, et al. A Phase 1 clinical trial of Hantaan virus and Puumala virus M-segment DNA vaccines for hemorrhagic fever with renal syndrome. Vaccine. 2012 Mar 2;30(11):1951-8.
16. Song JY, Woo HJ, Cheong HJ, Noh JY, Baek LJ, Kim WJ. Long-term immunogenicity and safety of inactivated Hantaan virus vaccine (Hantavax) in healthy adults. Vaccine. 2016 Mar 4;34(10):1289-95.
17. Song JY, Jeong HW, Yun JW, Lee J, Woo HJ, Bae JY, et al. Immunogenicity and safety of a modified three-dose priming and booster schedule for the Hantaan virus vaccine (Hantavax): A multi-center phase III clinical trial in healthy adults. Vaccine. 2020 Nov 25;38(50):8016-23.
18. Cho HW, Howard CR. Antibody responses in humans to an inactivated hantavirus vaccine (Hantavax). Vaccine. 1999 Jun 4;17(20-21):2569-75.
19. Sohn YM, Rho HO, Park MS, Kim JS, Summers PL. Primary humoral immune responses to formalin inactivated hemorrhagic fever with renal syndrome vaccine (Hantavax): consideration of active immunization in South Korea. Yonsei Med J. 2001 Jun;42(3):278-84.
